# Stoicism, mindfulness, and the brain: the empirical foundations of second-order desires

**DOI:** 10.3389/fpsyg.2025.1569237

**Published:** 2025-04-30

**Authors:** Marc Wittmann, Carlos Montemayor, Mauro Dorato

**Affiliations:** ^1^Institute for Frontier Areas of Psychology and Mental Health, Freiburg, Germany; ^2^Department of Philosophy, San Francisco State University, San Francisco, CA, United States; ^3^Department of Philosophy, Communication and Performing Arts, Roma Tre University, Rome, Italy

**Keywords:** free will, self-regulation, stoicism, Libet clock procedure, voluntary action, diachronic agency

## Abstract

Building on the Stoic notion of self-regulation, we explore philosophical conceptualizations in relation to empirical evidence from psychology and cognitive neuroscience. We challenge the mainstream account that dismisses the possibility of free will based on contemporary scientific findings. Instead, we argue that these findings actually support and refine the Stoic view of free will, particularly in terms of diachronic self-regulation through second-order willed actions over time. Contrary to classical interpretations of Libet-type experiments—which are often cited to refute free will—we contend that such evidence undermines the notion that we are passive recipients of spontaneous desires. Rather, we possess the capacity to regulate our actions proactively by cultivating and exercising deliberate, voluntary intentions. Freedom, in this sense, arises from a meta-cognitive ability or hierarchical, second-order will that can causally influence or override first-order desires or impulsive habits. In essence, our choices are not entirely predetermined by our upbringing or external circumstances; they emerge from our capacity to reflect upon and respond to those influences. Through this process, the self becomes a self-determined free agent.

## 1 Introduction

In recent decades, numerous scientists and philosophers have sought to convince the public that free choices are an illusion. We might subjectively experience the choice as dependent on our consciously willed decisions, but this feeling is illusory (Harris, 2012; Gray, 2015; Sapolsky, 2023; Krueger and Grüning, 2025). Their core claim is that our behavior and experiences are driven by numerous causes—both immediate and distant—which operate outside our awareness and beyond our conscious control. According to this view, decisions arise from unconscious processes, shaped by temporally proximal factors (like brain activation) or distal influences (such as genetic predispositions), and simply “happen” to us without any deliberate input from a conscious self. This perspective is perhaps most vividly captured by the metaphor of humans as fairground puppets or marionettes, controlled by unseen strings (Gray, 2015).

We disagree with this interpretation and contend that the scientific findings often cited to deny the possibility of free will do not, in fact, lead to this conclusion. While various authors have presented similar arguments against the view that free will is an illusion (see among others Dennett, 2004; Tse, 2013; Mele, 2014), our argument centers and develops the Stoic view of free will because such a notion enables us to clearly distinguish the notion of *choice* (a first-order, temporally located, causally *synchronic* event) from that of *free will* (a second-order *diachronic* trait, or an *evolving disposition*). As we take it, the core of the notion of the “Stoic view of free will” is based on a deterministic worldview in which, however, we are capable to control what depends on us (thoughts, emotional reactions to happenings, actions) by approving or rejecting all impressions caused by external events that do not depend on us. This control is a by-product of a constant practice and is realized in different degrees by human beings.^1^

Scientific findings, particularly in the field of cognitive neuroscience, do not rule out the existence of free will regarded in this way. Additionally, we demonstrate that the Stoic perspective already recognized free will not only in the capacity to choose and act but also as something that exists in degrees (see, for example, O’Connor, 2009; Keiserman, 2021). Nevertheless, this Stoic conception of free will requires updating, which we undertake in this paper to challenge the notion that free will is merely an illusion.

The conceptual framework presupposed by our paper centers on the opposition between the so-called compatibilism and libertarianism. As customary in the literature, we define compatibilism as the view that determinism – regarded as an ontological view stating that initial conditions and laws of nature fix a unique future – is compatible with free will. Libertarianism is the view that determinism so conceived is incompatible with free will and that in important circumstances of our life we could have willed to do otherwise.

In what follows we take for granted a minimalist compatibilist position, which holds that a free agent develops the ability to navigate determined first-order causal structures through a second-order perspective shaped over time and through habituation. While remaining within the framework of determinism, as the Stoics envisioned, this second-order structure cannot be reduced to the simpler network of stimulus–response relationships characteristic of first-order interactions, which the free agent learns to utilize. Since we intentionally set aside the metaphysical debate between compatibilism and libertarianism in this discussion, we do not aim to offer a definitive critique of libertarianism. Our perspective is a *hierarchical compatibilist* one, which not only affirms Frankfurt-style second-order desires but also highlights the cumulative influence of disciplined learning on the overall framework that governs freely willed action. This complex disposition, we argue, does not and need not rely on any form of *indeterministic* process. Depending on upbringing, specific encounters with books, other people, and various influencing factors, the self-regulation and discipline necessary and sufficient for freedom manifest differently in each individual. This empirical fact explains why, as the Stoics foresaw, humans do not possess freedom to the same degree. When we attribute Sapolsky and others the view that free will is an illusion we think that according to them not only is libertarianism wrong. We oppose Sapolsky’s hard determinism view by taking for granted the soft compatibilist view, according to which determinism and free will are compatible. Even if our actions, decisions and second-order desires are a consequence of previously determined events, we can do what we want and to a certain extent want what we want. We do not discuss the compatibilist-libertarian debate for the simple reason that we assume at the outset that compatibilism intended in this sense is the correct view and want to argue that we can defend it from the most common objection to it, namely concerning the interpretation of empirical results surrounding the Libet task.

## 2 Tracing the origins of the notion of free will

The notion of *free will* has been studied in detail in Western philosophy, a tradition in which its existence is central to ethics. Starting with Plato and Aristotle, Greek philosophers used terms concerning choice and responsibility, developed ethical systems, and had views about rationality. However, according to authoritative historians of Greek philosophy, both Plato and Aristotle lacked a notion of free will (Dihle, 1982). This is remarkable, given that moral responsibility is at the forefront of many of these authors’ writings (e.g., Plato’s *Republic* and *Timaeus,* particularly the Socratic notion that no one does willingly what is wrong; Aristotle’s *Eudemian Ethics*, *Nicomachean Ethics*, and *Politics*, where the notion of habituation and control in choosing is examined, a notion which influenced the Stoic concept of agential control). In particular, Aristotle was convinced that *action* was a decisive element to change our inclinations: by acting courageously (an act that depends on our second order will) we can become progressively courageous and therefore gradually overcome even a strong previous, first order tendency toward acting cowardly (Aristotle, 2020). Thus, it is important to be careful in defining which aspects of our notion of free will are fundamental for the capacity to be responsible and in control of our behavior, and how adequate the philosophical notion of free will is in understanding human psychology.

Our main goal is to develop an empirically adequate assessment of the problems of free will that captures the essential contributions of the origin of this concept, as articulated by the Stoic philosophers, and to offer an account of free will that follows the path that, to our knowledge, has been opened by them. Of course, our account does not intend to be a detailed reconstruction of the stoic view of free will within their moral philosophy (Sorabji, 2000; Brennan, 2003; De Harven, 2016; Salles, 2016). It will serve us as a guide to orient ourselves in the relevant but enormously vast empirical literature, with the aim of providing a strong argument against those who claim that we have no free will.

More in detail, we argue that contemporary *empirical* findings in psychology and neuroscience support the Stoic view in the sense: that free will is based on (1) our careful assessments of the *contents of experience*, (2) the ensuing second-order reactions to stimuli that create the development of evolving-in-time patterns of behavior rather than merely one-time responses to stimuli or contents.^2^ The current metaphysical debates seem to be based on the problem of establishing whether an unconnected series of synchronic, *single instances* of decisions or choices are free in a compatibilist sense or not, in particular with respect to “self-forming” ones (Kane, 1998; Balaguer, 2010) that are supposed to determine the kind of persons that we will become.

Following the Aristotelian insight that a diachronic control over time is necessary for responsible and rational agency, the Stoics argued that such control is the genuine manifestation of the free will in an agent who gradually learns to evaluate their immediate emotional and cognitive responses over time. Since this process typically involves prolonged training and development, the Stoic account is better equipped to engage with contemporary findings on free will than the prevailing focus in the literature on isolated decisions or choices made “one at a time.”

The historical debate on the notion of free will in ancient philosophy centers in part on when exactly such a notion first emerged, particularly in the West. There are very good reasons for tracing the emergence of the concept of “free will” back to Stoicism, particularly to the work of Epictetus (Frede, 2011, p. 76). The Stoics held that true freedom requires liberating ourselves from false beliefs, which are not grounded in reality, and from emotional attachments that are irrational. Only by doing so can we not only make genuine choices but also attain true freedom to act. Historical evidence suggests that the concept of freedom of the will is far more robust than the notions tied solely to choice and responsibility, as it was meaningful only within the fully deterministic and hierarchical framework of the Stoics (Frede, 2011, pp. 66–68). The notion of free will, in fact, was central for responsibility in the Stoic context because, unlike the work of Plato and Aristotle, it is committed to an extreme kind of determinism, required by the kind of habituation that we will examine below (see Bobzien, 1998). In Stoicism, freedom is a notion that applies to very few people, the wise ones, who are capable to create and pursue a pattern of behavior leading toward living a good life in the sense specified by their philosophy.

By elaborating on the stoic notion of “pronoia”^3^ also the Christian authors believed in divine determinism in the form of providence and omniscience, which was necessary to make some room for either the human freedom of the wise (as in Origen) or human wisdom as the result of divine grace (as in Augustine; Warner, 1963). Trying to establish how much of the ancient notion and its motivations remain in our modern conception of free will is beyond the present inquiry, but the connection to responsibility and ethics certainly remains crucial for our current understanding of free will. Psychological research shows to what extent free will beliefs are quantitatively related to moral responsibility beliefs (Stinnett et al., 2022). These are the very aspects of free will that, for instance, Sapolsky (2023) denies, on the basis of his interpretation of the scientific evidence. The deniers argue that deterministic causes are often accepted, yet from a moral perspective, individuals are expected to resist these causes by exercising a will that is itself uncaused—creating a logical paradox (Krueger and Grüning, 2025).

As is well-known, the contemporary debate about the possibility of free will is based on the modern notion of *deterministic laws of nature* that has replaced the problem of reconciling the stoic providence or God’s omniscience with the human responsibility or freedom of action. This may create the impression that our notion of a free will is, therefore, very different from the ancient notion. Here we will just provide two claims in order to dispel this suspicion. First, the kind of causal determinism defended by the Stoics is not conceptually different from the modern view that laws and initial conditions imply a unique, nomically necessary future course of event. According to the Stoics in fact, causation is necessitating: “This thesis follows from the combination of two ideas: (i) particular causal relations are subsumed under strict regularities and (ii) these regularities necessitate – in the same circumstances, the same effects have to obtain” (Salles, 2016, p. 19). Secondly, even Aristotle’s implausible concept of the *Unmoved Mover*, has been relied upon by contemporary philosophers in order to account for libertarian views about free will. For instance, Watson (1982) notion of “immanent cause,” or agent-specific causation, finds echoes in recent discussions of free will in neuroscience (Tononi et al., 2022).

The vast literature on the metaphysics of free will centers on issues regarding causation and determinism, and therefore on the debate between the compatibilist and incompatibilist views (Fischer et al., 2007; Vargas, 2013). To move forward, we need to reframe the debate by integrating insights into how the brain works, revisiting the Stoic concept of self-regulatory control, and rethinking causation through the lens of embodiment and emotions. This has been indeed the focus of recent research in the cognitive neuroscience literature.

## 3 The Libet paradigm

A major task of an empirically informed diachronic approach to free will is to address the seminal work of Libet et al. (1983), which spurred the notion that we do not have free will on neuroscientific grounds. Even though this work has been already widely discussed in the last four decades, our counterargument is somewhat original to the extent that it refers to the conception of freedom that we want to defend here, one stressing its ‘evolutionary’, process-like and diachronic aspect. Notably, Libet himself did not dismiss the concept of free will. He proposed that while volitional processes begin unconsciously, conscious awareness can ultimately influences the outcome of an action through a veto mechanism (Libet, 1999).

Historically, the neuroscientific argument against free will started with the discovery of the *readiness potential* (Bereitschaftspotential), a slow negative shift in the EEG signal that precedes the onset of spontaneous, self-initiated action (Kornhuber and Deecke, 1965). Libet et al. (1983) were able to show that the readiness potential (RP) occurred about 350 milliseconds before the conscious intention to move, thereby challenging the causal relevance of a choice by an agent at any specific time (for a summary of his research, see Libet, 1999). This preconscious brain activity, as it is supposed to be related to movement intention and later movement execution, is often interpreted as a proof that all voluntary acts are preceded and determined by neural processes that we cannot *voluntarily* control (Haggard, 2011). I want chocolate ice cream or not. I have no control over the decision. Similarly, a binary choice situation would be: I want chocolate ice cream instead of vanilla ice cream, but the decision has been made by the brain before I am aware of it. In two impressive studies on such binary choices like pressing a left or a right button (Soon et al., 2008) and adding or subtracting numbers (Soon et al., 2013), it was possible to relate slightly above chance – but statistically significantly – fMRI brain activity recorded several seconds before to participants’ reports of their conscious decisions to perform one of the two actions.

The interpretations concerning the research outcomes about the Libet task have led to conceptual criticism as well as new empirical insights (Brass et al., 2019). In essence, the latter invalidate the strong conclusions regarding the readiness potential as the preconscious cause of our willed actions. For a comprehensive overview of the ‘rise and fall’ of the readiness potential, see Neafsey (2021). Registered brain activation preceding conscious choices may reflect stages in the decision processes rather than determine its outcome. That is, these decision processes could be related to prepared reflexes which participants form at the beginning of the rather artificial experimental setting (Brass et al., 2019). Participants have to perform a repetition of dozens of the same choices concerning when (Libet et al., 1983) and which button (Soon et al., 2008) to press. In this situation of monotonously performing the same decision over and over again, brain activity may reflect automatic motor biases. The decision to participate voluntarily has been made at the beginning of the experiment; during the experiment decisions are conducted rather automatically. In these types of experiments, there is no particular reason why one would choose for the 20th time when and which button to press (Mele, 2014).

The question arises, whether the experiment is really about free will, when one carefully weighs options, or rather about pre-reflexive decision stages, for example, the *intention* to act, that obviously precedes the action. Stephan Schleim (2024, p. 95) in his comprehensive book *Science and Free Will* has pointed out that Benjamin Libet wisely limited his claim regarding the readiness potential (as the name implies) to the unconscious initiation of movements rather than the determination of acts of will. This crucial distinction was quickly overlooked in both scientific and media discussions. In addition, the kind of ‘actions’ that follow a decision to press a button in an experimental setting are radically different from those preceded by complex and long deliberations whose outcome can affect our entire life by shaping our future self (“self-forming actions” in Kane, 1998’s language; for these kinds of decisions, see also c). It is in such life-forming decisions that our freedom and responsibility are really at stake, since they are much more important to establish what kind of persons we are and want to become. Consequently, it has been noted that the corresponding choices seem rather different from ‘button pressing’ since the activity of deliberation is enormously more complex. A study by Maoz et al. (2019) explored the difference between deliberate, ecologically significant decisions and arbitrary choices. When participants selected which of two non-profit organizations would receive a $1,000 donation, no readiness potential (RP) was detected. However, when making an arbitrary decision between the two options using a button press—despite both organizations ultimately receiving an equal $500 donation—a clear readiness potential was observed. Complex life decisions often entail envisaging multiple possible courses of action and require not only probabilistic judgments but also the consideration of deeply held values and emotional investments. These factors must be carefully weighed when making comparisons. Considerations of factors such as specific historical moments, the influence of education, and similar elements help to provide a more complete picture. Since freedom of the will, in Kane’s (1998) sense, involves self-forming actions, the decisions that precede them can be regarded as free if and only if they reflect the *whole preceding, relevant history* of the self that makes them.

In this respect, the Stoic perspective briefly discussed above is extremely important because it clearly shows why any mono-causal explanation of an “instant-like” intention to act, even if it can be regarded as a genuine choice, is insufficient to establish the freedom of the will. In such a perspective, “becoming free” is a *temporal process* entailing extended periods of time that presupposes a self that is also extended in time: a disposition or a character trait developed by constant effort and *tension* that, unlike the Epicurean strive toward *distension*, is typical of the Stoics’ mindset (see Hadot, 2002).

Technically speaking, neurophysiological work has shown that the readiness potential is not a causal predecessor to a given individual intention to move but is *an averaging artifact across many trials of the experiment* (Jo et al., 2013), an interpretation that had been voiced early on already by Eccles (1985). That is, the readiness potential reflects the average of a gradual increase in neuronal activity across many trials (Schurger et al., 2012). In a given individual trial the brain potential is visible as positive or negative potential shifts over time (VaezMousavi and Barry, 1993). When participants decide to press the button, in around 2/3 of trials a negative potential is recorded; in 1/3 of trials, however, a positive potential is recorded, nevertheless, in trials with a decision to act (Jo et al., 2013; Schmidt et al., 2016). On average, because of this unequal ratio of negative and positive brain potential shifts, a negative curve is typically measured that precedes self-initiated movement. What is more, the readiness potential occurs also when subjects make automatic, unconscious movements (Keller and Heckhausen, 1990; Takashima et al., 2018).

The readiness potential is therefore likely related to unspecific preparatory processes (Alexander et al., 2016; Neafsey, 2021), to the build-up of a felt urge to move (Keller and Heckhausen, 1990; Jo et al., 2014; Schmidt et al., 2016), or to gradual decision-making processes preceding movement execution (Miller and Schwarz, 2014). Conscious *decisions are not made at an instant as the clock method* in the Libet task might imply, when a subject has to indicate the exact time of intention. “[I] ntention consciousness does not appear *instantaneously* but builds up progressively. In this view, early neural markers of decision outcome are not unconscious but simply reflect conscious goal evaluation stages which are not final yet” (Guggisberg and Mottaz, 2013, p. 1, our emphasis).

What is often forgotten in mono-causal explanations is that the readiness potential is just one of many brain responses recorded and filtered out of the EEG or MEG signal (Buzsaki, 2006). Regarding the whole spectrum of brain activation with different frequencies, many brain signals that are associated with voluntary decision-making do not precede but appear at the same time when intentions are felt. For example, event-related desynchronization of beta-band activity in the EEG appears directly before and when subjects report their decisions to move (Jo et al., 2016); gamma-band activation as recorded with MEG occurs at the same time with subjective intentions to press one of two response buttons (Guggisberg et al., 2008). A multitude of neural signals in unison relate to felt choices. In other words, the idea that there is a temporally preceding brain activation pattern (the cause) that later generates an instantaneous conscious intention (the effect) does not hold under neuroscientific scrutiny (Guggisberg and Mottaz, 2013; Schmidt et al., 2016; Neafsey, 2021).

## 4 The spectrum of free will: meta-cognitive self-regulation

In the following, we build upon the previously broached empirical data and philosophical conceptualizations to elucidate our arguments in favor of the view that not only in common sense parlance but according to science we have free will.

The first argument, inspired by the Stoic conception, is about a notion of *relative free will*, compatible with the diachronic components of a free will, or what we call *diachronic regulation*. As extreme sides of a continuous spectrum exhibiting larger or smaller degrees of freedom in choosing our actions, two exemplars of individuals may be positioned. On the one hand, we can think of a heroin addict who is in urgent need of the next shot. This person has the impulsive focus of attention on a sole target. He would be considered as minimally free, even if his conscious intention to get the shot causes his action to inject it. On the other hand, we can imagine a highly experienced meditator with a history of meditation practice spanning decades^4^ and extremely trained in cognitive and emotional self-regulation, independently of one-time actions. The heroin addict will hardly be able to abstain from getting the next shot when the drug is available; where circumstances allow, the experienced meditator will be able to choose among many more different envisioned options than the heroin addict. In between these two extremes, we find different gradations in our capacity to control ourselves in which *free will consists.*

It is our conviction that the capacity to make free choices presupposes and is entailed by the capacity for self-regulation, which is necessary and possibly sufficient for a free action. The sufficiency in question depends on an extremely difficult problem concerning the nature of the self. We can control relevant motions of our limbs, regulate or control our emotions, but finally, in order to be free, we must be capable of developing a self that can “regulate itself” by a complex activity of self-reflection. This is the key insight of the Stoic conception of a free will. In short, to the extent that selves can be regarded as complex entities constituted by values, memories, autobiographical traits, etc. we can regulate ourselves thanks to the overruling, self-reflective activity of an attentive self: in important moments of our lives we simply choose, as Frankfurt and Balaguer have already argued, *the kind of selves that we want to become*.

An essential aspect of the problem of free will therefore concerns the kind of emotions that we want to develop in order to act and realize what we care about the most. Emotions are the relevant means to discover the way in which we want to project ourselves in the future. The capacity to plan requires the ability to detach oneself at least in part from the present circumstances, and therefore to invest time and energy in building a consistent self across the future. The stress here is in the word *consistency*, given that irrational drives due to *akrasia* or weakness of the will compromise the stability and well-being of human beings. Consequently, as previously mentioned, we aim to avoid the age-old, yet in our view potentially misleading, debate surrounding the compatibility or incompatibility of free will with determinism. The capacity to acquire self-regulation of our emotions and actions ought to be plausibly regarded as requiring deterministic chains of events at all levels. But even if, as List (2019) suggests, it necessarily involves indeterministic choices on the part of agents, what we are asserting here is simply that such a capacity is, as confirmed by the empirical evidence discussed below, actually manifested and realized by humans, and potentially by other animals as well. What is relevant in developing the skill to choose or act freely is the existence of relevant educational institutions teaching us to learn to self-regulate.

In psychological terms, the spectrum of free will covers the endpoints of impulsivity (minimal free will) and emotional and cognitive self-regulation (maximum free will). We distinguish between a person who is impulsively present-oriented (lacking self-control) and a person who is mindful experiencing and acting in the present moment (having strong self-control) with a view about her future. There is a partly neglected distinction between self-regulation and self-control (Witowska et al., 2020) that here is extremely relevant. Self-regulation pertains to a form of “democratic,” internal “negotiatory” adjustment, which complies with one’s own internal values and needs. In contrast, self-control is a more authoritarian, repressive type of behavioral regulator. However, we use the terms self-control and self-regulation interchangeably. The impulsively oriented person has a strong urge to react to stimulation without considering later negative consequences; the mindful person is in an observational state associated with more self-regulation and is able to weigh the pros and cons of her behavior. Accordingly, it is not about absolute freedom of voluntary decision-making but about more or less freedom of choices. In a nutshell, the more consciously (or mindful) choices are made, the freer the individual (Schleim, 2024).

The concept of mindfulness, which has recently been established in Western psychology, is understood as bringing awareness to each present moment with an accepting and non-judgmental attitude, i.e., being especially aware of ongoing thoughts, perceptions, and emotions, a capacity which can be developed through meditation practices (Eberth and Sedlmeier, 2012; Lutz et al., 2009; Wittmann and Schmidt, 2013), and which seems essential to the kind of discipline, habituation, and consistency required by the Stoic conception of free will. Similarly to the mindfulness state of mind, such a discipline involves constant attention to our spontaneous actions and reactions to events. Through ongoing self-education via spiritual exercises (similar to meditative practices), which were central to Hellenistic philosophy and beyond, we can guide ourselves toward achieving a life of freedom. The term *spiritual* exercises may frighten the modern reader, tells us Hadot (2002). But “[T] hese exercises in fact correspond to a transformation of our vision of the world, and to a metamorphosis of our personality” (Hadot, 2002, pp. 81–82). Thus, the concept of ‘being mindful’ is a modern elaboration of the stoic doctrine (Pigliucci, 2015).

In scientific conceptualizations, being mindful is empirically correlated with increased *emotional self-regulation* capacities, leading to inhibitory control over inner impulses and immediate urges (Sauer et al., 2011; Bowlin and Baer, 2012; Wittmann et al., 2014). Since meditation practice can be described as deliberately and continuously focusing attention on the present moment, *attention regulation* as higher-order cognition is one of the further defining features of mindfulness (Bishop et al., 2004). Regular meditation practice enhances the ability of attention regulation in everyday states, i.e., having the capacity to continuously focus attention on a task without being distracted, thereby forming a persistent individual trait (Jha et al., 2007). The two aspects of attentional (cognitive) and emotional self-regulation define our idealized individual who is mindful in everyday life. Relatedly, we argue that the possibility of choosing freely *necessarily* requires mindful attentional states of this sort, even if the *reason why* we end up choosing one course of action over another were to entail a reliably deterministic chain concerning (1) the effectiveness of our action and (2) the effectiveness of the controlling and regulating part of our selves. This kind of training of attention in relation to freedom and control is relevant to virtue approaches in ethics and epistemology (Montemayor, 2023).

Impulsive behavior is defined as a reaction to a situation in the present moment without thinking about future consequences (Ainslie, 1975). Impulsivity is an extreme form of temporal short-sightedness, with the dominance of hedonistic present orientation and a lack of future orientation (Zimbardo and Boyd, 1999). More impulsive people exhibit typical short-sighted-behavior in daily life such as gambling, having unprotected sex, risky driving, or using drugs (Keough et al., 1999). As is well-known, such short-sightedness in decision-making has negative consequences in the long run as it affects life achievements (Mischel et al., 1989). For example, for children and adolescents, doing school homework has consequences only in the long run, while play time is immediately rewarding. In laboratory tasks, impulsive individuals more often choose smaller rewards obtainable after a shorter delay over larger rewards which are only obtainable after longer delays (Wittmann and Paulus, 2009). The urge to receive the smaller reward now is stronger than the incentive of the larger reward later. This is because waiting is associated with an emotional cost and more impulsive people feel more negatively when waiting and at the same time overestimate felt duration (Jokic et al., 2018). In contrast, more self-controlled individuals are relatively more likely to wait to receive the larger reward. Individuals with stronger emotion-regulation capacities feel less bored while waiting and time passes subjectively faster (Witowska et al., 2020). In general, more self-controlled individuals have the ability to override and refrain from their immediate desires and opt for more rational decisions (Baumeister et al., 2007). This is what we should expect if the Stoic claim about the importance of maintaining constant tension and focus in our minds, as a means to achieve inner freedom, is accurate.

In the mindfulness conceptualization (Kohls et al., 2009), individuals are highly aware of the ongoing changes in their thoughts, perceptions, and emotions. This involves two levels of cognition (Schooler, 2002; Jankowski and Holas, 2014): a basic level where one experiences thoughts, perceptions, and emotions in the present (knowledge), and a higher level of meta-awareness about these experiences (knowledge about one’s knowledge). Mindfulness meditation trains this meta-awareness, such as noticing a wandering mind during meditation and refocusing on the breath (Hasenkamp et al., 2012). In contrast, an impulsive individual experiences thoughts and emotions on the basic level, often on autopilot, while a mindful person recognizes themselves as the observer of these experiences. This meta-cognition allows the mindful person to be present and self-regulate cognitively and emotionally. People with better self-regulation are better equipped to cope with difficult situations using adaptive strategies (Mikulincer and Florian, 1997).

In our empirical approach, it is appropriate to reinterpret a well-known solution to the problem of free will presented by Frankfurt (1971), who has argued that we are free in the sense that the capacity for self-control is a meta-cognitive ability, or second-order will (a desire to desire), which is causally effective in enforcing and inhibiting first-order desires (for an ontological reading of this ability in terms of dispositions, see Dorato, 2022). Being free in this sense means having the ability to evaluate what matters most to us during critical moments in life, and to influence and guide our immediate desires in alignment with our deeper, second-order desires, ultimately shaping us into the person we aspire to become.

First-order willing would depend on the myriads of biological and cultural influences we are constantly exposed to. Consistent with the Stoic view, our freedom lies in effectively changing these dispositions through second-order control (self-regulation capacities), i.e., causally effective will-power. The famous dictum ascribed to Arthur Schopenhauer “a man is free to do what he wants, but not free to want what he wants” is the classic compatibilist stance.^5^ It means that, as long as nobody stops me with brute force or other impediments are present, I am free if I can do what I want. However, according to Schopenhauer, I cannot change what I actually want (I cannot want what I want).^6^ This could mean two things. Either (i) none has second-order desires or wills that can change what one wants. That would go against the Stoic view and the psychological conceptualizations of self-regulation capacities which we claim are part of what it means to be human. Or (ii) we do have these second-order desires but they are powerless in influencing our first order ones. It is obviously of decisive importance to establish *empirically* whether we have such a capacity or not.

Before tackling a more threatening conceptual objection (the infinite regress of will to will to will …) let us first note that, on an empirical level self-regulation happens in a short time frame of seconds as well as over longer-lasting processes of insight. On the short time frame, conscious cognitive appraisal processes modulate and regulate the activity of core emotional systems (Koelsch et al., 2015), i.e., the orbito-frontal cortex being a key neural structure in the selection and inhibition of other neural circuits associated with emotional responses (Rule et al., 2002). This intentional inhibition frees us from conducting immediate responses; it functions as a ‘neural brake’ in ongoing behavior and decision making which gives us the power of saying ‘no’ (Filevich et al., 2012). That is, emotional reactivity is always embedded in the self-regulatory appraisal of the entire situation. Relating to the aforementioned Libet-type of tasks, study results with an exceptionally experienced Buddhist meditator were similarly interpreted as showing a two-stage process of (1) sensing an inner impulse to act that was related to the readiness potential in the EEG and (2) a subsequent inner decision process related to the feeling of choice when initiating the act, or not (Jo et al., 2014). Thus, the constantly upcoming feelings and urges are modulated through conscious self-regulation activity. It goes without saying that in all of the above, we are referring to those frequent life situations in which we *can* wait before acting, not to situations in which we must act as rapidly as we can to avoid an immediate danger.

Though it may well be impossible in each circumstance to succeed in changing one’s first-order dispositions, what is essential to us is that trying to change them presupposes the existence of the corresponding disposition, or better, the desire to develop the disposition. Furthermore, there is empirical evidence suggesting that practicing the change of one’s disposition to behave in a certain way can lead to the development of a different, and potentially even opposite, disposition. Despite the obstacles of habit and automatic reactivity, in the course of our life we can also gradually change what we want over longer time frames. Insight into what we want to do (behavior) or who we would like to be (personality) can lead to gradual changes through intentional, free choices in life—choices that are consistent over time and reflect a persistent self, rather than a series of one-time decisions characteristic of a “disconnected” self without a clear life plan (Rawls, 1971).

The conceptual objection anticipated above^7^ consists in arguing that if we need to postulate a meta-meta will we generated an infinite regress, since it is wills all the way down. However, from a simple, hardly to be denied introspective viewpoint, when we evaluate whether, in an important circumstance, we ought to set aside more time to play with our children (first-order desire 1) or go abroad to a workshop that will definitely foster our carrier (first-order desire 2), we are caught in a moral dilemma characterized by the presence of *two* different (reasons) to act.^8^ During deliberations, second-order desires emerge, since we evaluate and weigh both desires in order to find out which is more conducive to become the kind of person that we want to become. After deliberation, we unearth or discover that we want (via a second-order desire) to reinforce, say, the will to become good parents by acting consistently with this desire, and thereby minimize future regrets. The whole deliberative process occurs in a soft compatibilist framework.

Although it was once thought that the personality would remain more or less stable after the end of adolescence, our individual traits change in middle and even later adulthood, as longitudinal studies show. This personality growth or maturation is reflected by the fact that on average individuals become less neurotic, more conscientious, and agreeable (Staudinger and Kunzmann, 2005). Gerontologists argue that wisdom in older age involves the balanced coordination of emotion, motivation, and thought (Staudinger, 2008). It has also been suggested that believing in free will could promote fitness-increasing behaviors, positively impacting health-related attitudes and behaviors, as well as leading to higher life satisfaction (Crescioni et al., 2016; St Quinton and Crescioni, 2022).

We would argue that it is not only the subjective belief that has positive consequences. Such positive changes over the life span are due to second-order will intervening in many important moments of our existence which is causally effective in shaping our first-order capacities for decision and choice. Over time we learn that we can control how to want what we want. It is important to note that, as a result of what we have discussed so far, it is only through a focused attention to our *present experience* that we develop the ability to project ourselves into the future. This is by no means a contradiction.

We can offer two arguments to defend this claim. The first is, as Augustine well knew (see Warner, 1963), given by the well-known fact that what we experience is always located in the present: we have *present* memories of the past, *present* experiences of the sensory world and *present* anticipations of the future. The second, related argument is that the way we live the present makes an enormous difference: living in the present or looking at the world from no particular temporal perspective (*sub specie aeternitatis* as Spinoza had it) are two different ways to experience the present that can be learned and that can in different ways free our mind from irrational emotions (see Dorato, 2021). In this sense, it is worth recalling the contrast between the ancient Epicurean and Stoic roads of wisdom as a way of realizing that, minding the present, is a way to be capable of controlling one’s natural dispositions (Hadot, 1995). The former insisted that we should concentrate on our present experience since our past and our future do not exist, and we only have a moment to live, which is the present moment. The uncertainty of the future and the awareness of having to die may render us more capable of minding the present by giving it the attention it deserves: rather than suffering from remorse or worrying about an uncertain future, we should seize the day:

“Inquire not […] how long a term of life the gods have granted to you or to me: While we are conversing, envious age has been flying; seize the present day, not giving the least credit to the succeeding one” (Horace, 1983, *Book 1*, *Ode 11*).

The latter is based on the awareness that the universe is regulated by unchangeable laws:

“[T]hink of how short is the span between birth and dissolution, and how vast the chasm of time before your birth, and how the span after your dissolution will likewise be infinite” (Quoted in Hadot, 1995, p. 183).

In both cases—whether by distending one’s mind in the present, as Epicurus suggests, or by exercising control over present emotions through understanding that they result from a long chain of events, as the Stoics teach—these are two distinct ways of interpreting the present experience. Both approaches liberate us from the traps of acquired but undesired dispositions. Marcus Aurelius, a key figure in the Stoic school, emphasized the significance of mindful concentration on the present moment with great intensity:

“[…] you cannot lose another life than the one you’re living now, or live another one than the one you’re losing…. The present is the same for everyone; its loss is the same for everyone; and it should be clear that a brief instant is all that is lost. For you can’t lose either the past or the future; how could you lose what you don’t have?” (Marcus Aurelius, 2006, p. 23).

## 5 Self-regulation as proactive willing

The reductive explanation (Harris, 2012; Sapolsky, 2023) for the illusory nature of free will probably stems from the mono-causal conception that there is brain activation at t_1_ (the cause) and after a certain delay a conscious intention (the effect) occurs at t_2_. Additionally, it is assumed that the neural signal at t_1_ causes the movement to occur at t_3_. This conception could be described as the billiard ball model. One could think that billiard ball 1 hits billiard ball 2, ball 1 thus causes ball 2 to move. The neural signal at t_1_ causes the conscious intention at t_2_. The billiard ball causality also happens between the events t_1_ and t_3_ on the neuro-behavioral level. We tried to show above that this conceptualization is refuted on empirical and theoretical grounds. There is not one single neural event that precedes one instantaneous conscious feeling and subsequent action.

Neural events are correlated with experience, an unfolding of neural processes accompanies the unfolding of an intention over time, but there is no temporal gap between a finalized neural process (say, the unconscious decision to move) and a later appearance of a subjective experience of the decision to move now. We do not adhere to a Cartesian-type dualism of the mind-brain problem but stay metaphysically neutral in this respect; subjective experience and neural events, are the 1st person and 3rd person perspectives, respectively, of decision making (Velmans, 2009). For example, there are neural correlates of decision making when a participant in a Libet-type study inhibits (to veto) an initial choice of acting (Brass and Haggard, 2007), such as in the dorsal fronto-median cortex and the left and right anterior insula. We also try to avoid the “double-subject fallacy” in which the brain and the person are treated as two independent subjects (Mudrik and Maoz, 2015). Such a misrepresentation happens in the literature on free will when scientists write about a brain event determining a decision which precedes a subject’s experience of the decision. In a similar way, Dennett and Kinsbourne (1992) argued against a cartesian theater model, according to which there is a place in the brain where “it all comes together” (t1) and subsequently (t2) is presented for subjective evaluation.

A second implication of the billiard ball model is that conscious awareness is a passive process which is at the receiving end of preceding neural causes. This Cartesian idea of experience being caused by external stimulation does not fit well with modern conceptualizations of the mind. Based on several ideas going back to von Helmholtz (1867, p. 430), perception is considered a predictive, goal-oriented process directed toward the future (Friston, 2010). Perception is not a passive sensory process (Eagleman, 2016). It relies on an active “predictive coding” processes about what is expected to happen. Perception is eventually a comparison between the prediction (what might happen) and actual sensory input (what happens).^9^ Phenomenal consciousness thus relies on constant short-term predictions and the subsequent prediction error, amounting to the difference between prediction and sensory input. Importantly, we do *not* necessarily adhere to the sort of internalism inherent in the predictive-coding conceptualization, where conscious experiences are fundamentally a brain-generated simulation or (controlled) hallucination (for a comprehensive critique of this kind of epiphenomenalism, see Hohwy, 2016; Zahavi, 2018).

What we want to highlight here is that the passive billiard ball model which is used to debunk free will is outdated. The predictive account of information processing presupposes that the brain and mind function proactively. Anticipatory mechanisms of experience are also found in the enactive and embodied conceptualizations of the mind which stipulate that we have direct access to the world (Thompson, 2007; Gallagher, 2017). Conscious perception thereafter is not generated by passive representational processes, but by sensorimotor activity of the proactively moving body as it dynamically assesses (has access to) the environment (O’Regan and Noë, 2001; Kirchhoff and Kiverstein, 2019). These approaches turn the classic stimulus–response logic around in that the world is apprehended through active search mechanisms, an internal generative model for anticipating and influencing upcoming events (Montemayor and Wittmann, 2022).

Anticipatory mechanisms of proactive agency fit the distinction between second-order and first-order will, where the second-order level affects the first-order level. While we desire many things (first-order), through the meta-level of desire (the desire to desire) we can self-regulate what we actually will do; we then accomplish what we “really” or genuinely want and turn our will into a free will, as the Stoics would put it. We do not necessarily self-regulate at a given present moment (self-causation at an instant in time) but meta-level values and goals set the criteria for future events. In the terminology of neuroscientist Peter Ulric Tse (2013) this is called *criterial causation*. According to him, we set criteria for upcoming events in advance (second-order, meta-level). In that sense, we are free to behave differently in the future, as we have set the criteria corresponding to our own values and goals: “Free will is fundamentally about the future, especially the next cycle of iterative information-processing loops” (Tse, 2013, p. 133). Now I am acting; thereafter I perceive a suboptimal outcome because I decided impulsively; next time I am confronted with a similar decision; I then adjust my choice; the more adequate outcome reinforces more optimal future decision-making. Through these future-oriented self-regulation processes I am capable and free to will what I will (or at least try to will what I will, that is, try to develop the kind of dispositions that lead me to become the kind of person that I want to be). Short-term self-regulation can happen in the range of seconds when I realize a mistake and immediately correct it; it could last decades in a process where someone reinterprets a long past event and acts differently the next time.

In experimental studies using computer tasks it has been shown that people sometimes feel that they are behaving in a certain way but actually are not doing so, e.g., stopping a cursor on a screen over an object when a human confederate of the experiment is causing the stop (Wegner, 2002). These findings of course only show that in certain circumstances we can be wrong in attributing conscious will, but we are nevertheless often quite right in our self-assessment (Mele, 2013). Moreover, the time scale of perception and behavior is important. When multiple events and actions unfold within a short period of time, in the range of seconds, as in Wegner’s experiments, a misattribution of the causes of actions and thoughts can indeed happen. On a longer time frame, freedom of choice is daily routine. We choose the seat in the plane with more leg space, and avoid certain foods or movies we do not like, etc. All of these choices are often based on higher-order criteria where we have learnt from former experience to adjust future behavior (Mele, 2014).

J.T. Ismael in her book with the programmatic title *How physics makes us free* (2016) adds to these deliberations on higher-order principles from a standpoint of physics. The mere fact that we can reflect (second-order level) on our potential decisions would make a deterministic equation (on the first-order level; stimulus–response) unsolvable (Ismael, 2016, p. 95f). Even if such a deterministic equation would in principle be possible, the self-feeding, self-regulating deliberations of an individual, who is thus self-determined with her values and beliefs, would make behavior unpredictable from a third-person perspective (e.g., a scientist), not only for differences between individuals but also within one person from one moment to the next (Ismael, 2016, p. 96).

It must be added that the radical unpredictability argued for by Ismael is compatible with an *ontic* determination of our action. Ismael sometimes ‘fuses’ determinism with predictability. She is, of course, aware that the unpredictability of our behavior—even to a Laplacean demon—does not imply that determinism, as an ontological concept, is false. In any case, the insertion of a reflection stage between stimulus and response creates an internal locus of control, a form of inner control of what is happening to us. There is no simple stimulus–response chain as behaviorists in the first half of the 20th century wanted to imply in their attempt to mimic the physical sciences (Strawson, 2019).

The neurophysiological processes that allow our choices to be shaped not just by upbringing and circumstances (the billiard ball model), but also by reflection on these factors (Ismael, 2016, p. 105), are now better understood. Empirical evidence points to the dorsal fronto-median cortex to be associated with voluntary inhibition of behavior which acts itself as an active causal factor (Kühn et al., 2009). These neuroimaging insights into the neural basis of action inhibition are consistent with our proposed view of self-control. Moreover, the proactive nature of the mind with its properties of predictive agency falsifies the simplistic stimulus-reaction model of behavior. It rather suggests that a human has self-regulation capacities to actively select his environment and accordingly change it to her needs. In that sense we as humans have free will.

## 6 Conclusion

Our goal was to develop an empirically sound assessment of free will, grounded in its origins as articulated by the Stoic philosophers, and to offer an account that builds on their foundational insights. Accordingly, our freedom lies in the ability to will to will (desire to desire). First-order willing is shaped by biological and cultural influences, while true freedom, as the Stoics held, depends on second-order self-regulation—exercising effective willpower to change these dispositions, that is, controlling our first-order desires. Complementing these philosophical ideas, research in psychology and cognitive neuroscience regarding the capacity for self-regulation shows we are free in the sense that we can evaluate what matters most in key life decisions and guide our first-order desires according to our second-order desires, shaping the person we want to become. The self continuously mediates between causes and effects, making it a self-determined free agent: Our choices are not merely determined by upbringing and circumstances; they emerge from thoughtful reflection on those influences. Despite the risks of relying on the contentious idea that Stoic foresaw later empirical discoveries, it is clear the Stoics had profound insights into this complex phenomenon—insights now supported by scientific evidence.

## Footnotes

^1^As Marcus Aurelius (2006, p. 33) aptly put it: “You have power over your mind—not outside events. Realize this, and you will find strength.”

^2^In the course of the paper it will become clear in what sense we develop and confirm from an empirical standpoint an important view that insists on second-order desires and that a few decades ago has been already expressed by Frankfurt (1971).

^3^A sort of divine and rational fate that governs the cyclic occurrence of events.

^4^Possibly, a meditator who has decided to become a monk in a monastery lacks free will because his motivation was to find himself in an environment with lesser freedom, stricter behavioral control with fewer choices. We are referring to an ideal but possible meditator with highly trained self-regulation capacities. On the other hand, it has been argued that living in a monastery can make someone free from obligations and stimulations in a complex world (Gray, 2015).

^5^The original sentence from his *Preisschrift über die Freiheit des Willens* (Schopenhauer, 1839/1978, pp. 58–59): “Du kannst thun was du willst: aber du kannst, in jedem gegebenen Augenblick deines Lebens, nur Ein Bestimmtes wollen und schlechterdings nicht Anderes, als dieses Eine.” “You can do what you will, but at any given moment of your life you can will only one definite thing and absolutely nothing else but this one thing.” (Prize Essay on the Freedom of the Will; translation by Eric F. J. Payne, Cambridge University Press, 1999, p. 21).

^6^Already Hobbes had argued that “Whether the will to do this, or not to do this, be in a man’s own election is plainly negative; that is to say, a man hath not election whether he will or no; for when he deliberates whether he shall do a thing or not do it, he does not deliberate whether he shall will or not will; to will is the act of the will itself, which is necessarily determined by the last appetite in deliberation” (Hobbes, 2017, p. 57). Other philosophers (Hume among these) embraced this compatibilist view. We use Schopenhauer’s formulation to introduce more effectively the view defended by Stoicism and by Frankfurt, namely that also the meta-will is free only in the soft deterministic sense, since also the second order will depends on previous causes.

^7^We thank one of the referees for having raised this important objection.

^8^This is an instance of a self- forming action in Kane’s sense (1998).

^9^The same principle reigns in deep neural networks.
